# Practical Notes on Diagnosis and Treatment

**Published:** 1910-08-27

**Authors:** 


### Practical Notes on Diagnosis and Treatment.

**Eczema of the Scalp.**

In a child, cut the hair short and soften the crusts with strips of flannel dipped in oil, and fasten on with a calico cap for six hours. After removal of the crusts an ointment of oleate of zinc or lead may be used, with later, perhaps, a few grains of ammoniated mercury added. Boric acid and starch poultices may be used for the preliminary cleansing, but linseed and bread poultices should be absolutely tabooed, as they too often serve as nutrient media for pus and other cocci.—*Dr. Radcliffe Crocker.*

**Amenorrhœa and Insanity.**

Insanity, especially melancholia, is frequently associated with amenorrhœa, and we may have to correct the impression of the patient's friends that the return of menstruation will necessarily be followed by mental improvement. It is true that if the mental condition improves menstruation usually returns, indicating not that the amenorrhœa is the cause of the insanity, but that nutritive conditions, which were probably responsible for both symptoms, have improved. Return of menstruation without mental improvement makes the prognosis of the insanity unfavourable.—*Mr. Bland Sutton.*

**Hæmoptysis.**

In the hæmoptysis of phthisis the use of circulatory depressants is desirable. From this point of view ipecacuanha is most serviceable. It should be given in small doses, repeated. It is not necessary nor desirable to produce emetic effects.—*Dr. R. W. Philip.*

**Prophylaxis of Aphasia.**

A suggestion worthy of consideration is that of attempting to develop a graphic centre on the right side of the brain by practising writing with the left hand. This practice must be persevered in until the movements of the left hand in writing have become instinctive as those of the right hand are. When this is the case there is good reason to believe that no lesion of the left side of the brain would deprive the patient of the power to write, and it is far from impossible that the power of speech would also be retained. Education of a graphic centre in the right side of the brain as a prophylactic measure is a very different matter from the same thing as a method of treatment commenced after hemiplegia has occurred, and in certain cases it might be worth while urging a patient to perfect himself in this accomplishment.—*Dr. F. C. Coley.*

